# Risk Factors for Radiation Necrosis and Local Recurrence after Proton Beam Therapy for Skull Base Chordoma or Chondrosarcoma

**DOI:** 10.3390/cancers15235687

**Published:** 2023-12-01

**Authors:** Mizuki Takahashi, Masashi Mizumoto, Yoshiko Oshiro, Hiroyoshi Kino, Hiroyoshi Akutsu, Kei Nakai, Taisuke Sumiya, Eiichi Ishikawa, Kazushi Maruo, Hideyuki Sakurai

**Affiliations:** 1 Department of Radiation Oncology, University of Tsukuba, Tsukuba 305-8575, Ibaraki, Japan; 2 Department of Radiation Oncology, Tsukuba Medical Center Hospital, Tsukuba 305-8558, Ibaraki, Japan; ooyoshiko@pmrc.tsukuba.ac.jp; 3 Department of Neurosurgery, Institute of Medicine, University of Tsukuba, Tsukuba 305-8575, Ibaraki, Japan; kino.h@md.tsukuba.ac.jp (H.K.); e-ishikawa@md.tsukuba.ac.jp (E.I.); 4 Department of Neurosurgery, Dokkyo Medical University, Mibu 321-0293, Tochigi, Japan; h-akutsu87@dokkyomed.ac.jp; 5 Department of Biostatistics, Institute of Medicine, University of Tsukuba, Tsukuba 305-8576, Ibaraki, Japan; maruo@md.tsukuba.ac.jp

**Keywords:** chordoma, chondrosarcoma, radiotherapy, proton beam therapy, brain necrosis

## Abstract

**Simple Summary:**

In this article, we retrospectively evaluate risk factors for local recurrence and radiation necrosis after PBT for 101 cases of skull base chordoma and chondrosarcoma. In multivariate analysis, chordoma and large tumor size were found to be significant factors for local recurrence and higher total dose was a significant factor for radiation necrosis.

**Abstract:**

[Proposal] Here, we retrospectively evaluate risk factors for radiation necrosis and local recurrence after PBT for skull base chordoma or chondrosarcoma. [Patients and Methods] We analyzed 101 patients who received PBT for skull base chordomas and chondrosarcomas from January 1989 to February 2021. Multivariable logistic regression models were applied for local recurrence, temporal lobe radiation necrosis rates, and temporal lobe radiation necrosis. [Results] In multivariate analysis, chordoma and large tumor size were independent significant factors for local recurrence. The 1-, 2-, 3-, 4- and 5-year local recurrence rates were 3.9%, 16.9%, 20.3%, 28.5% and 44.0% for chordoma and 0%, 0%, 0%, 0% and 7.1% for chondrosarcoma, respectively. The local recurrence rates of small tumors (<30 mm) were 4.3%, 14.7%, 17.7%, 17.7% and 25.9%, and those for large tumors were 3.6%, 15.1%, 19.2%, 32.7% and 59.6%, respectively. In multivariate analysis, BED Gy_10_ and total dose were risk factors for radiation necrosis. [Conclusions] For skull base chordoma and chondrosarcoma, the risk factors of local recurrence were chordoma and large tumor size, and those of radiation necrosis were BED Gy_10_ and total dose, respectively. DVH analysis is needed to investigate the risk factors for brain necrosis in more detail.

## 1. Introduction

Primary tumors at the base of the skull are rare. Skull base chordoma accounts for 0.5% of primary brain tumors and chondrosarcoma accounts for 0.1% in Japan [1]. The standard treatment for skull base chordoma or chondrosarcoma is tumor resection [2,3]. However, due to the complexity of the anatomy of the base of the skull, it is difficult to completely remove the tumor with a sufficient margin. Therefore, postoperative irradiation is performed to improve local control [4,5]. In photon radiotherapy, about 50 Gy is administered based on the tolerable dose of the surrounding brain and optic nerve, but the 5-year local control rate is still <50% [4,5,6]. Proton beam therapy (PBT) has better dose convergence than photon radiotherapy [7,8] and this characteristic of PBT results in good treatment outcomes [9,10,11,12]. At our hospital, we have obtained good local control of skull base chordoma using high-dose PBT of ≥70 Gy(RBE) in postoperative irradiation [13,14]. Adverse events from PBT are generally minor, but brain necrosis in the temporal lobe due to high-dose administration is occasionally observed [13,14,15]. Here, we retrospectively evaluate risk factors for radiation necrosis and local recurrence after PBT for skull base chordoma or chondrosarcoma.

## 2. Patients and Methods

The subjects were 101 patients (male 46, female 55; Table 1) who received PBT for skull base chordomas and chondrosarcomas at the University of Tsukuba Hospital from January 1989 to February 2021. The patients had a median age of 51 (range 5–78) years old; an ECOG performance status (PS) of 0 (*n* = 72), 1 (*n* = 27), 2 (*n* = 1) or 3 (*n* = 1); a pathological diagnosis of chordoma (*n* = 83) or chondrosarcoma (*n* = 18); a surgical approach of endonasal surgery (*n* = 75), others (*n* = 23) or unknown (*n* = 3); a surgical result of gross-subtotal resection (*n* = 61), partial resection (*n* = 32) or biopsy or no surgery (*n* = 8); initial treatment (yes = 84, no = 17) and a tumor maximum diameter of <30 mm (*n* = 51), 30–49 mm (*n* = 14), ≥50 mm (*n* = 15, or unknown (*n* = 21). Gross-subtotal resection was defined as ≥95% of the tumor resected and partial resection as <95% resected.

Hyperfractionated PBT was selected in 71 cases and the median total dose was 78.4 Gy(RBE) [60–95 Gy(RBE)]. Since 2006, a standard dose of 78.4 Gy(RBE) in 56 fractions of hyperfractionated (twice per day) irradiation has been used. The initial 58.8 Gy(RBE) consisted of GTV as the resection cavity and residual tumor, the CTV as GTV plus 5 to 10 mm margin along with the surgical pathway, and the PTV as CTV plus 1 to 5 mm margin. In this approach, the brainstem received a dose of 60 Gy, the optic chiasm received 50 Gy and the dose received by the retina was kept below 40 Gy. The remaining 60.2 to 78.4 Gy(RBE) focused on the residual tumor and resection cavity. The same treatment protocol was used for chordomas and chondrosarcomas. Dose distribution was calculated using a broad beam algorithm (VQA, Hitachi Ltd., Ibaraki, Japan). The median dose per fraction (fr) was 1.4 Gy(RBE) [1.1–3.0 Gy(RBE)] and a twice-per-day irradiation method was selected in 71 cases. The median dose per irradiation was 2.5 Gy(RBE) [1.8–3.0 Gy(RBE)] for once-daily irradiation and 1.4 Gy(RBE) [1.1–1.4 Gy(RBE)] for twice-daily irradiation. Irradiation methods were not standardized initially. Until about 2006, irradiation was performed once a day with a total dose of about 72 Gy(RBE) [13]. Since 2006, irradiation has been performed twice a day, and administration of 78.4 Gy(RBE) in 56 fr (2 fr per day) has been widely used [14].

Local recurrence and temporal lobe radiation necrosis rates were estimated using the Kaplan–Meier method. The cumulative incidence for local recurrence with the competing risk of death without local recurrence was estimated with the ordinary nonparametric method. Multivariable logistic regression models were applied for local recurrence and temporal lobe radiation necrosis rates, and a multivariable Fine–Gray regression model [15] was also used for local recurrence and temporal lobe radiation necrosis. The candidate covariates in these models were gender, age (≤50 vs. >50 y), PS (0 vs. 1–3), initial treatment (yes vs. no), tumor maximum diameter (<30 vs. ≥30 mm), surgical approach (endonasal surgery vs. others), results of surgery (gross-subtotal resection vs. partial resection, biopsy or no surgery), pathology (chordoma vs. chondrosarcoma), total dose (<78.4 vs. ≥78.4 Gy(RBE)), dose per fraction (≤2 vs. >2 Gy(RBE)) and BED (biologically effective dose) Gy_2_ (≤135 vs. >135 Gy(RBE), BEDGy_10_ (≤89.5 vs. >89.5 Gy(RBE)) and hyperfractionated), where BEDGy_2_ = nd(1 + d/2) (n = irradiated fraction, d = dose per fraction). Variable selection for multivariable models was conducted using the stepwise method with AIC [16,17,18,19,20]. Multiple imputations based on the MICE method were applied to the missing covariates [21,22]. The significant level for statistical tests was set at 0.05 and the confidence level for confidence intervals was set at 95%. Analyses were conducted using the R software v.4.2.0 (R Core Team, Vienna, Austria). The grades of brain necrosis were defined as follows: Grade 1, imaging findings only/asymptomatic; Grade 2, mild symptoms/medical treatment only; Grade 3, severe symptoms/needs surgical treatment; Grade 4, life-threatening symptoms; Grade 5, death due to necrosis (CTCAE Version 5.0: central nervous system necrosis). The radiation necrosis was diagnosed using multiple contrast-enhanced MRI scans taken at least one month apart, and Methionine PET was also performed as needed. Every outpatient follow-up was conducted by radiation oncologists and neurosurgeons. The final decision was confirmed during a weekly conference of the department of radiation oncology and neurosurgery. Essentially, asymptomatic brain necrosis was managed through follow-up only. If symptoms occurred or if the lesion showed continued growth on the MRI images, steroid administration was initiated. In this study, surgical resection was not performed, and 2 patients received steroids.

## 3. Results

**Figure 1:**
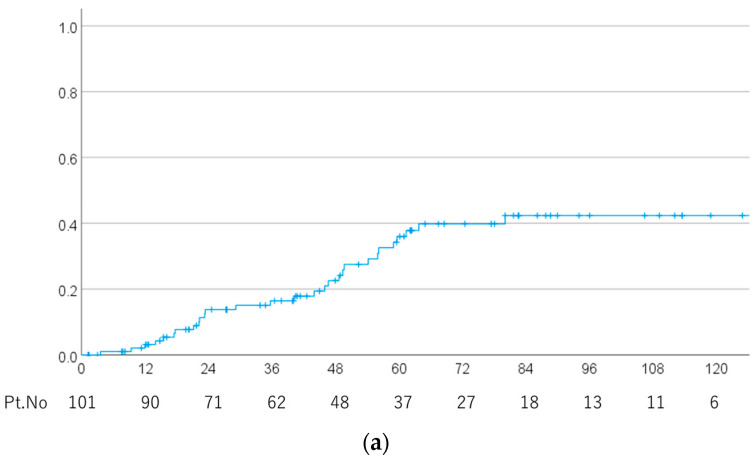

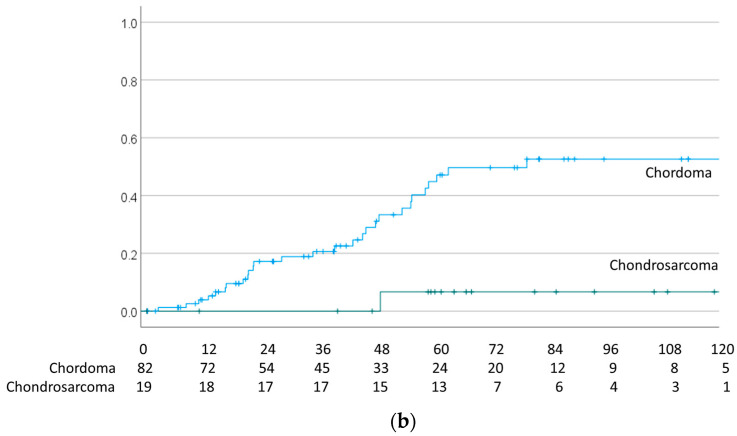
(**a**) Local recurrence rate in all patients. (**b**) Local recurrence rates for cases of chordoma and chondrosarcoma.

At the last follow-up, 19 patients had died and 82 were alive. The median follow-up period for survivors was 59.5 (range 1.3–191.8) months. At the last follow-up, 29 patients had local recurrence (Figure 1a) and the 1-, 2-, 3-, 4- and 5-year local recurrence rates were 3.2% (95% CI 0–6.7%), 13.8% (6.5–21.1%), 16.4% (8.6–24.2%), 22.6% (13.2–32.0%) and 36.0% (24.6–47.4%), respectively. Of these 29 patients, 15 underwent surgery for recurrence and had recurrence confirmed pathologically. In the other cases, recurrence was confirmed through at least two imaging examinations. The candidate covariates in univariate and multivariate analysis were gender, age (≤50 vs. >50 y), PS (0 vs. 1–3), initial treatment (yes vs. no), tumor maximum diameter (<30 vs. ≥30 mm), surgical approach (EES vs. others), results of surgery (gross-subtotal resection vs. partial resection, biopsy or no surgery), pathology (chordoma vs. chondrosarcoma), total dose (<78.4 vs. ≥78.4 Gy(RBE)), dose per fraction (≤2 vs. >2 Gy(RBE)) and BEDGy_2_ (≤135 vs. >135 Gy(RBE), BEDGy_10_ (≤89.5 vs. >89.5 Gy(RBE)) and hyperfractionated). In univariate analysis (Table 2) initial treatment, pre-treatment tumor maximum diameter and pathology were significant. In multivariate analysis (Table 3), pathology and pre-treatment tumor maximum diameter remained as independent significant factors. A multivariate analysis performed in chordoma cases only indicated that tumor maximum diameter was the only risk factor for local recurrence.

**Figure 2:**
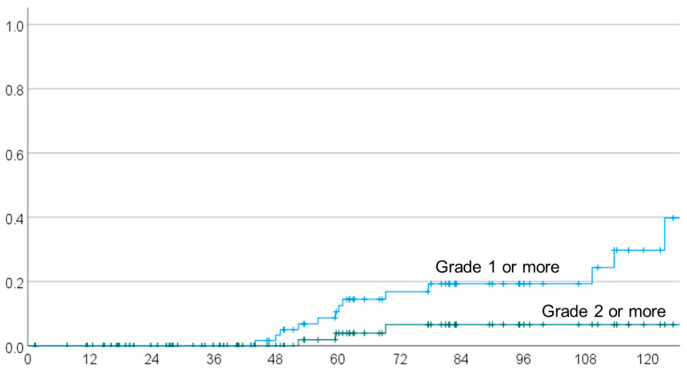
Rate of temporal lobe radiation necrosis in all patients.

The 1-, 2-, 3-, 4- and 5-year local recurrence rates were 3.9% (95% CI 0–8.2%), 16.9% (8.1–25.7%), 20.3% (10.7–29.9%), 28.5% (16.9–40.1%) and 44.0% (30.5–57.5%) for chordoma and 0%, 0%, 0%, 0% and 7.1% (0–20.6%) for chondrosarcoma, respectively (Figure 1b). The local recurrence rates of small tumors (<30 mm) were 4.3% (0–10.2%), 14.7% (3.7–25.7%), 17.7% (5.5–29.9%), 17.7% (5.5–29.9%) and 25.9% (10.6–41.2%), and those for large tumors were 3.6% (0–10.5%), 15.1% (1.4–28.8%), 19.2% (4.1–34.3%), 32.7% (13.9–51.5%) and 59.6% (39.4–79.8%), respectively. At the last follow-up, brain necrosis was observed in 13 patients: Grades 1 to 5 in 10, 2, 0, 0 and 1 cases, respectively. The absence of a continued increase in brain necrosis was confirmed on at least two MRIs. The Grade 5 case was diagnosed as cerebral necrosis based on the judgement of the attending physician, but the details were unavailable because it was an old case. The 1-, 2-, 3-, 4- and 5-year overall brain necrosis rates were 0%, 0%, 0%, 3.3% (95% CI 0–7.8%) and 10.6% (2.6–18.6%), respectively, and those for Grade 2 or higher (Figure 2) were 0%, 0%, 0%, 0% and 3.9% (0–9.2%), respectively. The covariates in univariate and multivariate analyses were the same as those used for analysis of local recurrence. In univariate analysis, dose per fraction, hyperfractionated and BEDGy_2_ were significant factors (Table 4). In multivariate analysis (Table 3), BED Gy_10_ and total dose were the risk factors for radiation necrosis.

**Figure 3:**
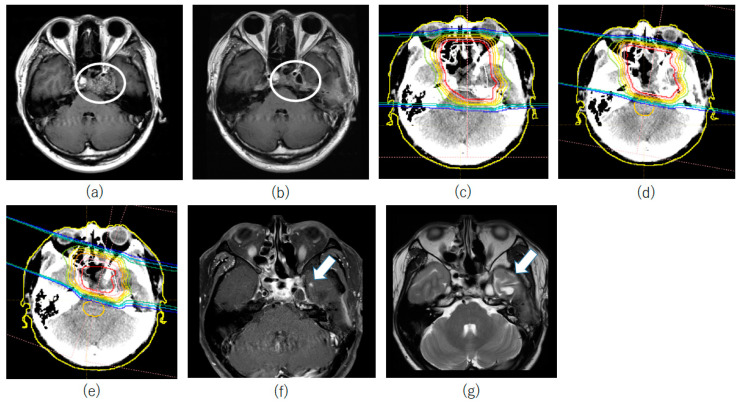
A case of temporal lobe radiation necrosis. (**a**) A tumor was found in contact with the left temporal lobe (white circle). (**b**) The tumor was grossly excised (white circle). (**c**) Irradiated field 0 to 39.2 Gy(RBE) in 28 fr (twice per day) including the tumor bed and surgical pathway. Dose distribution. The isodose lines represent 100–10% of the prescription dose from inside to outside. (**d**) Irradiated field 40.6 to 58.8 Gy(RBE) in 42 fr (twice per day) including the tumor bed and surgical pathway with a minimized radiation dose to the optic nerve, optic chiasm, temporal lobe, retina and brainstem. (**e**) Irradiated field 60.2 to 78.4 Gy(RBE) in 56 fr (twice per day) with a minimized radiation dose to the optic nerve, optic chiasm, temporal lobe, retina and brainstem. The maximum dose to the retina is 50 Gy(RBE), that to the optic nerve and chiasm is 54 Gy(RBE) and that to the brainstem and temporal lobe is 60 Gy(RBE). (**f**) At 44 months after proton beam therapy, contrast-enhanced MRI showed a small contrast-enhanced lesion in the left medial temporal lobe (white arrow). This spot was included in the irradiated area at boost. (**g**) There was mild edema around the contrast-enhanced lesion (white arrow).

To illustrate our findings, we present a case with necrosis of the temporal lobe. The patient was a 33-year-old male with chondrosarcoma on the skull base abutting the left temporal lobe. The patient underwent nasal lumpectomy (Figure 3a), and the tumor was completely grossly excised (Figure 3b). Two months after surgery, postoperative PBT was started. Initially, 39.2 Gy(RBE) in 28 fr (twice per day) was applied to a range including the surgical path and surgical cavity (Figure 3c). Subsequently, the brainstem, optic nerve, optic chiasm, retina and temporal lobe were dosed down, up to 58.8 Gy(RBE) in 44 fr (Figure 3d). Finally, the irradiation range was limited to the resection cavity, and irradiation up to 78.4 Gy(RBE) in 56 fr was performed (Figure 3e). Later, 44 months after PBT, contrast-enhanced MRI revealed a small contrast-enhanced lesion in the left medial temporal lobe (Figure 3f), with mild edema around this lesion (Figure 3g). The site of the lesion was clinically determined to be cerebral necrosis because it coincided with the irradiation range of the final boost. There were no subjective symptoms due to brain necrosis in the medial temporal lobe. At 78 months after PBT, contrast-enhanced and edematous lesions have shown no tendency to progress and there are still no subjective symptoms.

## 4. Discussion

Surgical resection is the first choice for skull base chordom a or chondrosarcoma, but complete resection is difficult. Many studies have suggested that adjuvant radiotherapy can improve the control rate of skull base chordoma that is substantially resected or in which the resection margin is not clean [23,24,25], and radiotherapy is used in about half of patients [1]. Dose escalation is thought to be an important factor for favorable local control: McDonald et al. suggested that ≥74.5 Gy was a significant prognostic factor [26]. Therefore, in photon radiotherapy, the stereotactic technique is often used for treatment of skull base chordoma, but the 5-year control rate is still about 50% or less [4,5,6,27,28,29].

In contrast, favorable results have been obtained using particle radiotherapy. Mizoe et al. obtained a 5-year local control rate of 85% using carbon ion radiotherapy and Takagi et al. found the same rate of 85% using carbon ion radiotherapy or PBT [30,31]. In radiotherapy, dose is a significant factor for local control, with Koto et al. identifying irradiation volume (GTV < 34.7 cc) [11] and Chubei et al. finding age, tumor maximum diameter, surgery, primary site, and tumor stage as significant factors for local control [32]. Our study suggested a 5-year local control rate of 64% for all tumors, which is consistent with previous reports. In multivariate analysis, tumor maximum diameter and pathology were significant factors associated with local recurrence. Previous studies have shown that chordoma has more local recurrence than chondrosarcoma [3,13,14]. In a multicenter study of Gamma-knife treatment of 16 Gy (median) for 51 postoperative (*n* = 30) or recurrent (*n* = 21, 41%) chondrosarcomas, Kawashima et al. achieved good 3-, 5- and 10-year post-treatment local control rates of 87%, 78% and 67%, respectively [33]. In our data, the 5-year local control rate was 92.9%, indicating a better outcome. The better results with PBT may be due to the higher irradiation dose, the margin of 5 to 10 mm to the tumor, and the high rate of first-line treatment and total-subtotal resection cases. However, there are only a small number of chondrosarcoma cases in the current study and an accurate comparison with previous results is difficult.

The influence of tumor maximum diameter on local recurrence seems to be valid based on past reports and the results of radiotherapy for other tumors [34,35]. In our analysis, total dose was not a significant factor for local control. This is probably because most dose fractions used were >70 Gy and even the lower dose fractions were sufficient to obtain local control. However, an escalated dose to the skull base leads to risks of brain necrosis or vision loss [36], and several reports have suggested necrosis of the temporal lobe in particular. Gordon et al. treated 31 patients with chordoma or chondrosarcoma with PBT and found 2 cases with Grade 2 or higher temporal lobe necrosis [37]. Similarly, Pehlivan et al. used PBT in 62 patients with basilar chordoma or chondrosarcoma and found 7 cases with temporal lobe necrosis, of which 2 were Grade 2 or higher [38]. Ares et al. treated 64 cases of skull-base chordoma and chondrosarcoma with spot scan PBT, of which 2 developed optic nerve nephropathy and 2 developed brain necrosis, but no risk factors were predictive of high-grade late toxicity [39]. In carbon ion radiotherapy for 33 patients with skull base tumors with high total and fraction doses, Koto et al. observed >Grade 2 brain injury in 9% of the cases, and suggested that the brain volume receiving >50 Gy(RBE) in 16 fr was a risk factor for brain injury [40]. A BED of 50 Gy(RBE) in 16 fr is almost 133 Gy (α/β = 2), whereas we used 78.4 Gy in 56 fr twice a day in 55% patients. A BED of 78.4 Gy in 56 fr once a day is calculated to be 133 Gy; therefore, the BED of our fractionation is larger than 133 Gy. In our study, the rates of >Grade 2 and >Grade 3 brain necrosis were 3% and 1%, respectively, and BED (α/β = 10) and total dose were significant risk factors, with 89.5 Gy and 78.4 Gy considered as a cut-off value. In univariable analysis, fraction size, hyperfractionation and surgical status were significant factors. These results suggest that hyperfractionation with a dose of 78.4 Gy in 56 fr twice a day is safe and useful to control skull base chordoma and chondrosarcoma, while reducing brain necrosis.

McDonald et al. used PBT for base-of-the-skull chordoma, chondrosarcoma, adenoid cystic carcinoma and malignant sinus tumors, and found that a high-dose volume (≥60 Gy for ≥5.5 cc of the temporal lobe, or 70 Gy for ≥1.7 cc in the temporal lobe) was a risk factor for temporal lobe brain necrosis [41]. In a study of brain parenchymal and temporal lobe DVH after PBT for skull base tumors, Pehlivan et al. found no significant thresholds for patients without adverse events in the temporal lobe and those with Grade 1 toxicities [38]. In addition, there was no significant difference between patients with no toxicity and those with Grade 3 or higher toxicity, but there was a significant difference between cases with grades 1 and 3 toxicity, using the generalized equivalent uniform dose (gEUD). Reports of radiotherapy for brain tumors have also generally shown that the risk of brain necrosis is significantly related to the dose and volume of normal brain irradiated [42,43,44,45]. In a study of 117 brain metastases in 83 patients treated with five-fraction stereotactic radiosurgery (SRS), Andruska et al. found a cumulative radiation brain necrosis incidence of 15% after a median follow-up of 10.3 months, with a median time to radiation brain necrosis of 6.9 months [42]. It was concluded that brain V25 > 16 cm^3^ (HR: 11.7 [1.47–93.3]) and brain V30 > 10 cm^3^ (HR: 7.08 [1.52–33.0]) were associated with a significantly higher risk of radiation brain necrosis. In dosimetric analysis of radiation-induced brainstem necrosis in 479 patients with nasopharyngeal carcinoma treated with intensity modulated radiation therapy (IMRT), Fan et al. found an incidence of brain necrosis of 1.25% (6/479) and a median time to brain necrosis after treatment of 28.5 (range 18–48) months. An evaluation of the dosimetric parameters (Dmax, the maximum dose; D0.1cc, the maximum average dose delivered to a 0.1-cc volume, and D1cc, D2cc, D3cc, D5cc, D10cc and Dmean, the mean dose) indicated that Dmax was the most significant predictive dosimetric factor for brain necrosis and suggested that the dose to the brainstem should be limited to Dmax < 69.59 Gy [43]. In a study of 388 patients who underwent SRS, Kerschbaumer et al. found that 15.7% developed radiation necrosis after a median period of 8 months, and concluded that a larger tumor diameter and higher radiation dose were associated with an increased risk of radiation necrosis, independently of tumor type [44]. Our results are similar to these findings. In a review of 51 reports focused on brain necrosis after SRS for arteriovenous malformations or brain metastasis, Milano et al. concluded that in single-fraction SRS for brain metastases, volumes receiving 12 Gy (V12), including target volumes of 5 cc, 10 cc, or >15 cc, were associated with risks of symptomatic brain necrosis of about 10%, 15%, and 20%, respectively [45]. For three-fraction SRS for brain metastases, normal brain tissue V18 < 30 cc and V23 < 7 cc were associated with <10% risk of brain necrosis. Finally, we evaluate the results of this study based on the latest reports [14,21,22,23,31,46,47,48,49,50,51,52,53,54,55,56,57,58,59,60,61]. According to the latest reports, the 5-year local recurrence rate for chordoma was about 15 to 35%, while for chondrosarcoma, it was less than 10%. In our analysis, the 5-year local recurrence rate of chordoma is slightly high at 40%, which is believed to be due to the low GTR rate in the 1900s. In fact, our recent 5-year local recurrence rate for chordoma is 25%, which is almost in line with the latest literature [14]. Regarding temporal lobe cerebral necrosis, Grade 3 or higher is considered to be 10% or less. There is significant variation among reports, but some studies indicate that asymptomatic temporal lobe disorder occurred in about 40–50% of cases, at the maximum estimate. To mitigate the risk of temporal lobe injury, it is necessary to clarify the relationship between irradiation dose and volume and the occurrence of temporal lobe injury through DVH analysis.

## 5. Conclusions

In our analysis, BEDGy10 and total dose were the factors related to the incidence of brain necrosis. In past reports, a high dose has been identified as a risk factor for cerebral necrosis, and thus, the present analysis yielded reproducible results. In our patients, ≥Grade 2 radiation necrosis only occurred in 3 of 101 cases. Given that almost all patients received PBT of ≥70 Gy, the rate of temporal lobe necrosis can be viewed as low. We note that this low incidence of brain necrosis of ≥Grade 2 may reflect the relatively short median observation period of about 4 years. Also, the PBT policy at our hospital is to minimize the dose to the brain and brainstem after ≥60 Gy(RBE). We also note that DVH analysis could not be performed because many cases were treated at a time when only the irradiation dose was analyzed, and an analysis including the irradiation volume could not be performed. In the future, we plan to extract only data that can be analyzed using DVH and evaluate the relationships among brain necrosis, irradiation dose and irradiation volume in more detail. Within these limitations, we conclude that tumor maximum diameter is a risk factor for local recurrence and that BEDGy_10_ and total dose are a similarly risk factors for temporal lobe radiation necrosis. DVH analysis is needed to investigate the risk factors for brain necrosis in more detail.

## Figures and Tables

**Table 1:** Characteristics of the patients.

Characteristics	Number	%
Age (years)		5–78 (median 51)
Gender		
Male	46	46%
Female	55	54%
Initial treatment		
Yes	84	83%
No	17	17%
ECOG performance status		
0	72	71%
1	27	27%
2	1	1%
3	1	1%
Surgical approach		
Endonasal surgery	75	74%
Others	23	23%
Unknown	3	3%
Surgical result		
Gross-subtotal resection	61	60%
Partial resection	32	32%
Biopsy or non-resection	8	8%
Pathological findings		
Chordoma	83	82%
Condrosarcoma	18	18%
Tumor maximum diameter (mm)		0–90 (median 20)
<30	51	50%
30–49	14	14%
≥50	15	15%
Total dose (GyE)		
<78.4 GyE	44	44%
≥78.4 GyE	57	56%
Dose per fraction (GyE)		
≤2 GyE	82	81%
≥2 GyE	19	19%
Hyperfractionated		
Yes	71	70%
No	30	30%
Biological effective dose (α/β = 2)		
<135 GyE	80	79%
135 GyE or more	21	21%
Biological effective dose (α/β = 10)		
<89.5 GyE	96	95%
89.5 GyE or more	5	5%

ECOG, Eastern Cooperative Oncology Group.

**Table 2:** Univariable analysis of potential predictive factors for local recurrence.

Factors	PT Number	5-Year (%)	Mean (Months)	95% CI	*p*-Value
Age (years)					
≤50	50	30.1	133.9	109.3–158.5	
>50	51	41.3	100.9	81.8–119.9	0.439
Sex					
Male	46	34.0	140.2	117.3–163.0	
Female	55	49.8	97.1	75.2–119.1	0.050
Performance status					
0	72	33.8	133.1	113.7–152.5	
1–3	29	41.8	83.8	58.6–109.0	0.137
Initial treatment					
No	17	61.2	62.0	31.8–92.2	
Yes	84	29.1	134.6	116.1–153.1	0.001
Tumor maximum diameter (mm)					
<30	51	25.9	110.8	93.7–128.0	
≥30	29	59.6	96.5	70.2–122.8	0.025
Surgical approach					
Endonasal surgery	75	37.4	118.9	97.3–140.4	
Others	23	31.8	110.2	88.2–132.3	0.365
Result of surgery					
Biopsy or partial	40	42.4	110.9	85.3–136.5	
Gross-subtotal resection	61	31.1	117.7	100.4–134.9	0.242
Pathology					
Chordoma	83	44.0	111.5	91.4–131.6	
Chondrosarcoma	18	7.1	136.5	123.9–149.1	0.006
Total dose (Gy)					
<78.4	44	45.4	112.2	85.5–138.8	
≥78.4	57	38.0	121.6	102.7–140.4	0.133
Dose per fraction					
≤2	82	29.2	109.5	93.2–125.9	
>2	19	20.3	138.3	98.0–178.7	0.445
Hyperfractionated					
Yes	71	33.2	117.1	99.9–134.3	
No	30	40.9	113.3	80.7–145.9	0.242
BEDGy_2_					
>135	80	36.0	112.2	95.8–128.7	
≤135	21	31.6	123.9	84.1–163.8	0.990
BEDGy_10_					
>89.5	96	35.4	112.4	97.2–127.6	
≤89.5	5	46.7	115.3	42.2–188.4	0.739

**Table 3:** Multivariable analysis of potential predictive factors for radiation necrosis and local recurrence. All (a) and chordoma only (b).

**(a)**			
**Factors**	**Odds Ratio**	**OR Range**	***p*-Value**
Radiation Necrosis			
BEDGy_10_	1.211	1.037–1.414	0.016
Total dose	0.111	0.065–1.290	0.005
Pathology	3.587	0.790–16.292	0.097
Gender	0.290	0.065–1.290	0.103
Local recurrence			
Pathology	0.115	0.013–0.988	0.049
Tumor maximum diameter	4.354	1.487–12.746	0.008
**(b)**			
**Factors**	**Odds Ratio**	**OR Range**	***p*-Value**
Radiation Necrosis			
BEDGy_10_	1.293	1.002–1.670	0.049
Surgical approach	6.540	0.584–73.221	0.126
Total dose	0.070	0.008–0.592	0.015
Age	1.070	1.001–1.144	0.046
Local recurrence			
Tumor maximum diameter	3.842	1.296–11.395	0.016

**Table 4:** Univariable analysis of potential predictive factors for radiation necrosis.

Factors	PT Number	5-Year (%)	Mean (Months)	95% CI	*p*-Value
Age (years)					
≤50	50	7.1	163.0	139.8–186.3	
>50	51	14.3	118.6	97.6–139.5	0.308
Sex					
Male	46	12.6	141.0	113.1–169.0	
Female	55	8.0	161.4	139.1–183.8	0.274
Performance status					
0	72	8.9	151.7	129.0–173.8	
1–3	29	17.9	119.3	96.2–142.4	0.613
Initial treatment					
No	17	25.0	121.5	84.5–158.4	
Yes	84	9.7	149.5	128.1–171.0	0.894
Tumor maximum diameter (mm)					
<30	51	24.4	128.5	97.5–159.5	
≥30	29	3.8	160.7	133.9–187.5	0.072
Surgical approach					
Endonasal surgery	75	12.9	140.6	113.9–167.2	
Others	23	5.3	167.6	143.1–192.0	0.179
Results of surgery					
Biopsy or partial	40	3.3	158.9	133.2–184.5	
Gross-subtotal resection	61	17.7	123.0	101.8–144.3	0.154
Pathology					
Chordoma	83	9.4	157.0	134.6–179.4	
Chondrosarcoma	18	13.8	110.9	89.1–132.7	0.146
Total dose (Gy)					
<78.4	44	22.1	145.0	117.6–172.5	
≥78.4	57	0.0	136.5	116.3–156.7	0.374
Dose per fraction					
≤2	82	4.5	144.7	130.9–158.6	
>2	19	30.2	117.7	81.5–153.9	0.011
Hyperfractionated					
Yes	71		144.2	128.7–159.7	
No	30		127.1	94.5–159.7	0.027
BEDGy_2_					
<135	80	4.6	143.7	129.0–158.3	
≥135	21	28.0	122.3	87.1–157.5	0.026
BEDGy_10_					
<89.5	96	13.3	134.6	118.7–150.5	
≥89.5	5	50.0	88.1	31.7–144.4	0.016

## Data Availability

All data are available on request to the corresponding author.
